# The impact of the education program based on dimensions of quality of work life among emergency medical services providers

**DOI:** 10.1186/s12913-024-10610-2

**Published:** 2024-02-28

**Authors:** Ali Panahi-Qoloub, Sima Zohari Anboohi, Malihe Nasiri, Parvaneh Vasli

**Affiliations:** 1 Student Research Commitee, Department of Medical-Surgical Nursing, School of Nursing and Midwifery, Shahid Beheshti University of Medical Sciences, Tehran, Iran; 2 Department of Medical-Surgical Nursing, School of Nursing and Midwifery, Shahid Beheshti University of Medical Sciences, Tehran, Iran; 3 Department of Basic Sciences, School of Nursing and Midwifery, Shahid Beheshti University of Medical Sciences, Tehran, Iran; 4 Department of Community Health Nursing, School of Nursing and Midwifery, Shahid Beheshti University of Medical Sciences, Vali Asr Ave., Ayatollah Hashemi-Rafsanjani Cross Road, Tehran, Iran

**Keywords:** Quality of work life, Emergency medical services, Education

## Abstract

**Background:**

Quality of work life is a vital factor for health care providers. This study aimed to determine the impact of the education program based on dimensions of quality of work life among emergency medical services employees.

**Methods:**

The quasi-experimental study was conducted on 100 emergency medical services employees in Tehran, Iran, who were chosen using a convenience sampling method (50 in the intervention group and 50 in the control group). The information was gathered using a Demographic Questionnaire and a Walton Quality of Work Life Questionnaire with eight dimensions. The research was carried out in three stages: design, implementation, and evaluation of the education program. During the design phase, the educational needs of the participants were determined in terms of the dimensions of the quality of work life and work and total living space. The education program on work-life quality was implemented in four virtual group sessions, emphasizing the educational needs identified through uploading educational content to the WhatsApp application. The evaluation was conducted in two stages: before the education program and three months after the program. With a significance level of 0.05, the data was analyzed using SPSS version 24 software.

**Results:**

The results revealed that an education program on the quality of work life and its dimensions, emphasizing strategies to improve work and total living space, can improve the score of this dimension in the intra-group comparison of both the intervention and control groups (*p* = 0.046), as well as in the inter-group comparison, at the three-month post-intervention stage, there is a significant difference and a significant increase (*p* = 0.030), but it does not have a significant effect on the quality of work life and its other dimensions.

**Conclusion:**

It is recommended that emergency medical services managers plan to improve the quality of working life of their employees, particularly in terms of work and total living space.

## Introduction

Emergency Medical Services” (EMS) means the planning, delivering services, care, and supervising emergency care by the personnel, organizations, and associations engaged in injured people [1]. EMS employees provide services in a hazardous environment regarding safety, resources, and equipment; as a result, they are exposed to numerous physical and mental health risks [2].

The Iranian EMS system was founded in 1975 and offers free services across the country under the supervision of the Ministry of Health and Medical Education [3]. The clinical setting for such services is offered in 3000 centers (viz., 1700 road centers, 1300 urban centers, and 50 Air medical emergency centers) [4]. Clients call “115” and speak with the emergency medical dispatcher, who takes a history and the caller’s address. The emergency medical dispatcher gives this information to a nearby base if a dispatch is deemed necessary. Emergency medical technicians evaluate the patient at the scene and may consult a physician in the dispatch center to determine whether the patient needs transport to a hospital. The emergency medical technicians then coordinate with the hospitals before arrival. There are 216 ambulance bases in Tehran, most with one ambulance and one motorcycle ambulance. Based on the dispatch call, one emergency medical technician drives the motorcycle ambulance to scenes where transport is not predicted to be necessary. The emergency medical technicians may provide limited medical care. A few bases have two ambulances, and a few have an ambulance bus, which is used for multiple casualties when air transport is limited. All bases are managed by one dispatch center [5].

All emergency medical dispatchers are nurses with bachelor’s degrees or physicians [5]. The emergency medical providers in bases are emergency medical technicians and paramedics. Emergency medical technicians evaluate patients’ conditions, provide them with primary medical care, and transport them to the hospital. Basic emergency medical technicians have a high school diploma and a basic life support certification for healthcare providers. They can drive the ambulance and help transport patients to the hospital. Intermediate emergency medical technicians have an associate degree and can evaluate patients’ conditions, provide primary medical care, and transport them to the hospital. Paramedics are healthcare professionals with bachelors in emergency medicine, nursing, and anesthesiology. They provide advanced health care. At least one EMS provider is employed as each ambulance’s intermediate or paramedic provider.

Stress is one of the most significant psychological risks for these employees. They are subjected to various stressful events, such as unexpected situations, suffering, pain, and death [6].

Stress and burnout can reduce the quality of work life (QWL) [7]. QWL is regarded as critical for healthcare-related organizations seeking to improve patient and employee satisfaction while also providing high-quality healthcare services [8]. QWL is a humanizing approach to a workplace regarding job nature, physical work environment, social connections, compensation and benefits, administrative approaches, and employee relations [9]. QWL has a multi-dimensional structure and includes ideas like welfare and health services, incentive plans, job suitability, job security, the importance of the individual’s role in the organization, ensuring growth and development, participation in decision-making, reducing job conflicts and uncertainties, an education and rewards system, and a person’s overall evaluation of his job [7, 10]. This study used the Walton model of QWL as a conceptual framework. It is possible to identify the positive and negative aspects affecting QWL using the Walton model (1973), and it has been demonstrated that in this model, the possible dimensions of QWL are combined [11]. QWL, he claims, can be explained in the following eight dimensions: (1) Fair and adequate compensation (the sufficiency of the salary received compared to the recipient’s subjective or set standards); (2) Safety and health in working conditions (employees should not be subjected to working conditions that harm their physical and mental health, including noise, lighting, workspace, avoiding accidents, and reasonable working hours appropriate); (3) Career opportunities and security (certainty about the job); (4) Constitutionalism in the organization of work (employee rights and responsibilities such as privacy, freedom of expression, egalitarian behavior, and rules and routines); (5) Social relevance of life at work (adequate image of the organization obtained through social responsibility and its benefits to society); (6) Work and total living space (employees’ experiences in the organization, which can impact their personal and social lives; 7) Social integration at work (providing an analysis of employee cohesion among themselves and within the organization based on proportional and balanced coexistence, and with the absence of prejudice, evaluates social support, hierarchical differences, and lack of bias among employees); 8) Opportunity for use and capacity development (the ability to use human skills immediately) [12].

QWL is a complex concept influenced by various factors in both work and personal life. QWL is influenced by physical and emotional well-being, economic conditions, personal beliefs, and cooperation in the workplace [13]. According to research on QWL in health services, factors such as increased workload and stress levels, inappropriate working hours, non-participation in decision-making, poor supervisory relationships, shift work and lack of opportunities to develop new skills, lack of facilities welfare for nurses, inability to balance work and family needs, insufficient number of employees, inadequate holidays, management and supervision methods, lack of professional development opportunities, lack of patient care supplies and equipment and recreational facilities all have a negative impact on QWL [8].

QWL is one of the most critical factors that can shape employees’ organizational behavior [14] and is a measure for developing organizational skills that provide three factors: satisfaction and motivation, responsibility, and commitment to work [15]. High QWL is required to achieve high performance, increase organizational profitability, and continue to attract and retain employees. Employees tend to stay in a job when they are satisfied and leave when they are not satisfied. It also impacts employee organizational commitment and the quality of healthcare services [16].

Education and empowerment can improve people’s QWL at work by giving them more control over their workload, making them feel more supported by colleagues, rewarded for their accomplishments, and treated fairly [17]. Improving the QWL of EMS employees has been identified as essential in ensuring the sustainability of the healthcare system. Despite being one of the most numerous work groups, very few studies have focused on the nature of their work and QWL [15]. Khabbazi Ravandi et al. investigated the impact of training components of QWL on improving it in an Iranian welfare organization [18]. Another study examined the effect of a QWL improvement program and the enrichment of marital life education on the QWL and job satisfaction of dual-career couples in Ilam, Iran [19]. Hashemi and Ghazanfari conducted a cross-sectional study on the relationship between occupational stress and QWL in EMS technicians [20]. Aminizadeh et al. surveyed to determine the relationship between QWL and the organizational commitment of Iranian EMS employees during the Coronavirus outbreak in Kerman, Iran [15].

Documenting and addressing QWL among health care staff is critical because lower QWL in this group can jeopardize patient care quality [7]. This study aimed to determine the impact of the education program (EP) on QWL among emergency medical services providers.

## Methods

### Study design and setting

This study used a quasi-experimental with two groups. Quasi-experiments are studies that aim to assess interventions, but they do not use randomization. These studies can use pre- and post-intervention measurements as well as non-randomly selected control groups. Some quasi-experimental designs with pretests use control groups for comparisons. Obtaining pretest measurements on both the intervention and control groups allows one to assess the initial comparability of the groups. The assumption is that if the intervention and the control groups are similar at the pretest, the likelihood of important confounding variables differing between the two groups is less significant [21]. Accordingly, the study used the intervention group (IG) and the control group (CG). The study’s environment included all urban-road EMS operational bases west and north of Tehran.

### Participants and recruitment

Based on Khabbazi Ravandi et al.‘s study in Iran [18] and taking into account the type I error of 0.05, the type II error of 0.10, the study power of 0.90 with a dropout rate of 10%, a total of 50 people in the IG and CG were considered.

$$n \ge 2\frac{{{{({z_{\alpha /2}} + {z_\beta })}^2}{\sigma ^2}}}{{{{({\mu _1} - {\mu _2})}^2}}}$$

Participants in the IG were drawn from the EMS bases in West Tehran, while those in the CG were drawn from the EMS bases in North-West Tehran via convenience sampling. The purpose of convenience sampling is to collect information from participants who are easily accessible to the researcher. The main assumption associated with this sampling is that the members of the target population are homogeneous. There would be no difference in the research results obtained from a random sample or a sample gathered in some inaccessible part of the population [22]. There are 43 EMS operational bases in West Tehran and 21 in North-West Tehran, with an average of six people working in different categories at each base. The researchers’ access to the participants was the reason for selecting participants from among the databases mentioned. It should be noted that all EMS providers in Iran are currently male.

**Fig. 1:**
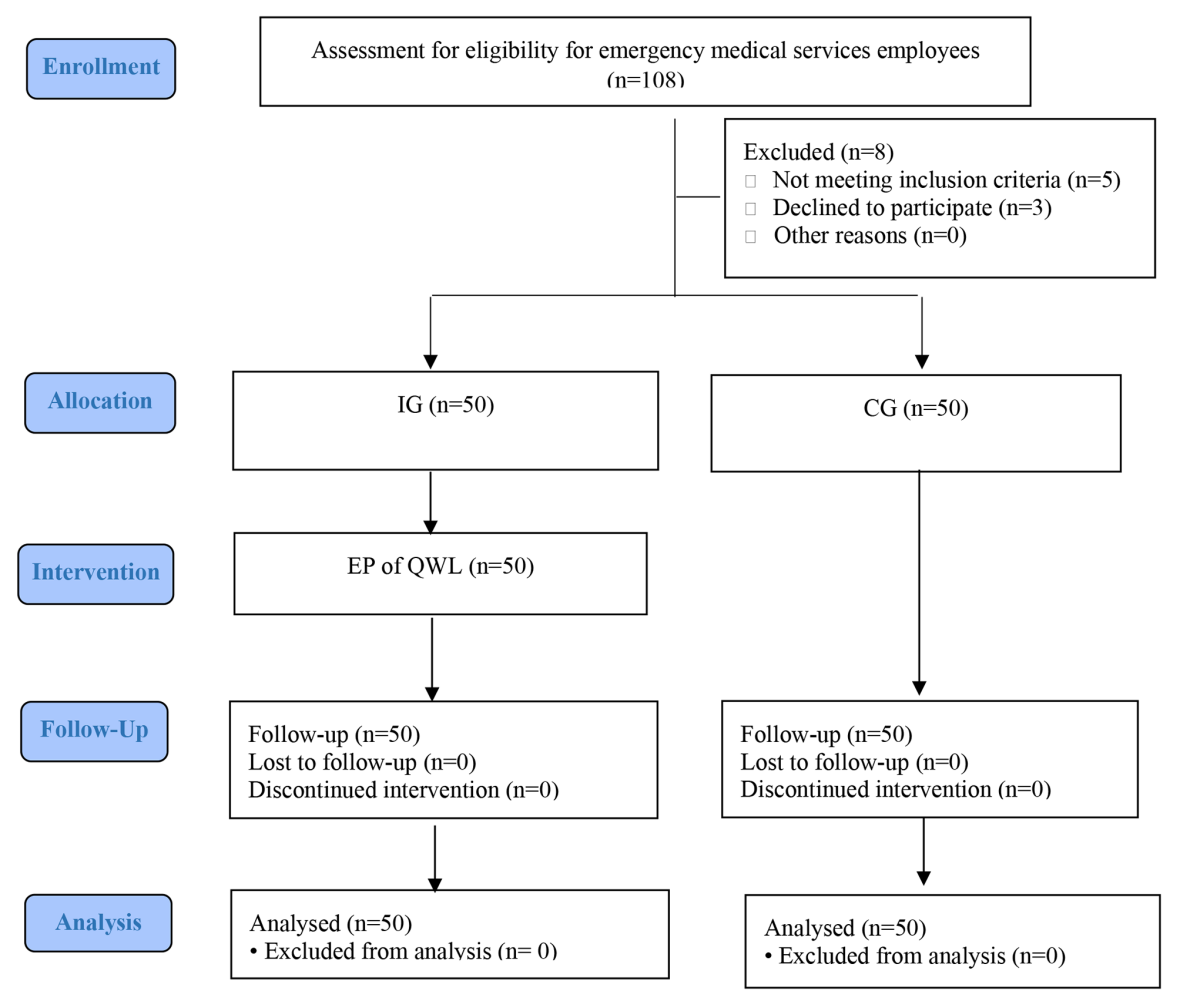
CONSORT flow chart of the study

The second researcher appeared in the research setting in the morning and evening of different days and, after explaining the purpose of the study, invited eligible people to participate. It should be noted that IG and CG had similar working conditions regarding hours, shifts, salaries, and benefits. Working as an operational force, working in EMS operational bases, and having at least six months of work experience in EMS were the inclusion criteria for the participants. Absence from training sessions is also an exclusion criterion. Four CG and six IG participants completed the questionnaire incompletely during the study, which was considered missing data. The exact number of other participants replaced them (Fig. 1).

### Measures

### Demographic questionnaire

Age, work experience, marital status, education level, field of study, employment status, income adequacy, and service bases were all questions on the demographic questionnaire.

### Walton QWL questionnaire

The Walton QWL Questionnaire was used in this study to collect data on QWL. This questionnaire was based on Walton’s factors (1973) and was designed by da Siva Tomossi et al. in 2008 [23]. The 35-item tool has been translated into Persian, and its psychoanalytic indicators have been assessed [24]. This 35-item tool has eight dimensions, including the following: fair and adequate compensation (4 items), safety and health in the working conditions (6 items), career opportunities and security (4 items), constitutionalism in work organization (4 items), social relevance of life at work (5 items), work and total living space (3 items), social integration at work (4 items), and opportunity for use and capacity development (5 items). The questionnaire is scored on a Likert scale ranging from very low (one score) to very high (five scores). A high score indicates that you have a better QWL. This questionnaire has a minimum score of 35 and a maximum score of 175 [25, 26]. Previous studies among Iranian hospital healthcare workers have examined the psychometric properties of this instrument, confirming its validity and reporting a reliability of 0.94 using the Cronbach’s alpha test [27]. The Cronbach’s alpha of the tool based on the responses of 20 EMS providers who were not included in the study was calculated to 0.88, which showed the instrument was reliable [28, 29].

### Design stage

The educational needs of EMS providers from Tehran’s West and North-West bases were determined at this stage. The demographic and Walton QWL Questionnaires were distributed to all research participants and asked them to complete and return to the researcher (baseline). After verification, the results obtained for each dimension in IG and CG are shown in Table 1. According to the researchers, dimensions with scores lower than their attainable mean should be considered a priority educational need to improve QWL. As the table results show, work and total living space have a lower mean score than the rest of the dimensions (compared to the mean value that can be obtained); thus, in the content, while taking the concept of QWL and its dimensions into account, the methods improvement of work and total living space were emphasized.

**Table 1:** The results of the evaluating educational needs of EMS employees about the QWL dimensions

QWL Dimensions	Achievable values			Mean ± Standard deviation in IG
	Min	Max	Middle	
Fair and adequate compensation	5	25	10	10.38 ± 2.09
Safety and health in working conditions	3	15	6	6.04 ± 2.33
Career opportunities and security	3	15	6	7.31 ± 2.13
Constitutionalism in the organization of work	6	30	12	13.52 ± 3.85
Social relevance of life at work	3	15	6	6.92 ± 2.20
Work and total living space	5	25	10	8.43 ± 3.01
Social integration at work	4	20	8	9.49 ± 2.93
Opportunity for use and capacity development	3	15	6	6.99 ± 2.30

*EMS*, emergency medical services; QLW, quality of life work; IG, intervention group

### Implementation stage

At this stage, the educational content was created using the available resources and implemented in accordance with Table 2 [30, 31]. Job burnout, stress management, time management, healthy lifestyle, the importance of family, friends, and colleagues, and how to communicate effectively with them were among the guidelines included in this educational series as strategies for improving work and total living space. In four sessions, the training was carried out through the formation of WhatsApp groups and the uploading of materials in the form of text and PowerPoint. The study used the WhatsApp app for virtual training for two reasons. The first reason is the interest and familiarity of Iranians with the WhatsApp application and its user-friendly features. The second was the lack of access to quality Persian-based free educational platform.

**Table 2:** EP of QWL dimensions with emphasizing ways to improve work and total living space

Session	Objective	Content
1	Getting to know the researcher and the objective of the research Getting to know the EP Getting to know the concept of QWL and its dimensions	− An introduction to the researcher, the title of the study and the process of conducting training sessions − Definition of QWL − Dimensions of QWL
2	QWL improvement strategies with emphasis on work and total living space	− A review of the content of the previous sessions − Job Burnout − Stress management
3	QWL improvement strategies with emphasis on work and total living space	− A review of the content of the previous sessions − Time management − Healthy lifestyle including nutrition and exercise
4	QWL improvement strategies with emphasis on work and total living space	− A review of the content of the previous sessions − The importance of family and friends, as well as how to interact with them effectively

*EP*, Educational program; *QLW*, quality of life work

Initially, the first researcher created a group on WhatsApp Messenger and added all the intervention group participants. After coordinating with the group’s participants on the day and time of the virtual meeting, the first researcher uploaded the educational content he had prepared in the form of videos and PowerPoint on four consecutive days. Participants could discuss and ask questions during and after the training session. At the end of each session, the summary of the training content was also provided to the participants of the intervention group in a pamphlet form. Participants who could not attend the class could benefit from the uploaded video and PowerPoint. In all days of educational material presentation, question and answer was used as one of the primary methods of learning.

### Data collection

Data were collected in two stages: before the EP of QWL (baseline) and three months after the EP of QWL (follow-up). The baseline data was collected after distributing the instruments to the participants of the groups. The Walton QWL Questionnaire was distributed virtually to IG and CG participants for follow-up. They were asked to complete and return it to the researcher in a WhatsApp chat.

### Data analysis

Descriptive statistics (mean, standard deviation, and frequency percentage) and inferential statistics were used for data analysis. Independent t-tests, chi-square test, Mann–Whitney U test and Fisher exact test were used to compare the demographic characteristics of IG and CG participants. Regarding QWL and its dimensions, the independent t-test was used for inter-group comparison, and the paired t-test was used for intra-group comparison. The data was analyzed with SPSS version 23 software and a 95% confidence level.

## Results

### Characteristics of the participants

The mean and standard deviation of age among IG and CG participants were 37.40 ± 0.91 and 37.72 ± 0.89, respectively. Furthermore, in IG and CG, the mean and standard deviation of work experience were 13.89 ± 0.81 and 14.15 ± 0.80, respectively. In terms of age and work experience, there were no significant differences between the two groups (*p* = 0.834 and *p* = 0.816, respectively). Table 3 contains additional demographic information.

**Table 3:** Demographic information of the participants in IG and CG

Group Variables	IG (*n* = 79)		CG (*n* = 79)			p-value
	M ± SD		M ± SD			
**Age**	16.8 ± 1.48		15.83 ± 1.68			0.27^a^
	**Frequency**	**Percentage**		**Frequency**	**Percentage**	
**Marital status**						
Married	40	80		38	76	0.510^b^
Single	10	20		12	24	
**Education**						
Diploma	2	4		1	2	0.444^c^
Associate	12	24		18	36	
Bachelor	35	70		30	60	
Master	1	2		1	2	
**Major**						0.553^b^
Emergency medical technician/ Responder	37	74		33	66	
Nursing	4	8		6	12	
Anesthesiology	9	18		11	22	
**Occupational status**						
Permeant	40	80		38	76	0.651^d^
contract	8	16		11	22	
Two-year recruitment	2	4		1	2	
**Monthly income**						0.620^d^
Inadequate	49	98		50	100	
Adequate	1	2		0	0	
**Prehospital emergency services**						0.589^d^
Urban emergency bases	47	94		49	98	
Road urban emergency bases	0	0		0	0	
Both	3	6		1	2	

Note: ^a^ Independent t-tests t-test, ^b^ Chi-square test, ^c^ Mann–Whitney U test, ^d^ Fisher’s exact test

*IG*, Intervention group; *CG*, control group

### QWL and its dimensions

Table 4 shows the results of a comparison of the participants’ scores on QWL and its dimensions. According to the findings, the mean QWL score in IG before and after EP was 71.62 ± 12.61 and 70.63 ± 12.73, respectively, and the mean QWL score in CG before and after EP was 71.22 ± 14.02 and 73.27 ± 13.67, respectively. The intra-group comparison results in both IG and CG did not show a statistically significant difference in the stages before and after EP (*p* = 0.849 and *p* = 0.212, respectively). Also, similar results were obtained in the inter-group comparison, with no significant difference in IG and CG in both stages of data collection (*p* = 0.208 and *p* = 0.054). These findings revealed that QWL training focusing on ways to improve work and total living space was unable to change the QWL score and its other dimensions significantly; however, in the case of some dimensions such as career opportunities and security, as well as constitutionalism in the organization of work, there was a significant difference in CG scores in the stages before and after EP (***p*** = 0.032, and *p* = 0.033 respectively). Still, this difference was not significant in IG. In addition, except for work and total living space, there were no significant differences in the stages before and after EP in the inter-group comparison.

**Table 4:** Comparison of changes of the QWL and its dimensions in IG and CG

Group QWL and its dimensions		IG	CG	p-value ^b^
		Mean ± Standard deviation	Mean ± Standard deviation	
Fair and adequate compensation	Baseline	10.80 ± 2.14	9.96 ± 2.04	0.061
	Follow-up	10.42 ± 1.91	9.82 ± 2.15	0.250
	P-value ^a^	0.020	0.070	
Safety and health in working conditions	Baseline	6.41 ± 2.41	6.39 ± 2.25	0.973
	Follow-up	6.61 ± 2.23	6.30 ± 2024	0.394
	P-value ^a^	0.516	0.052	
Career opportunities and security	Baseline	7.29 ± 2.22	7.34 ± 2.04	0.882
	Follow-up	7.42 ± 2.09	7.18 ± 2.01	0.462
	P-value ^a^	0.600	0.032	
Constitutionalism in the organization of work	Baseline	13.8 ± 3.97	13.24 ± 3.74	0.879
	Follow-up	13.29 ± 4.05	12.96 ± 3.81	0.599
	P-value ^a^	0.682	0.033	
Social relevance of life at work	Baseline	6.89 ± 2.30	6.95 ± 2.11	0.857
	Follow-up	6.92 ± 2.32	6.84 ± 2.11	0.802
	P-value ^a^	0.903	0.095	
Work and total living space	Baseline	8.21 ± 2.99	8.65 ± 3.04	0.874
	Follow-up	11.29 ± 3.93	8.05 ± 3.02	0.030
	P-value ^a^	0.046	0.052	
Social integration at work	Baseline	9.44 ± 3.00	9.54 ± 2.86	0.827
	Follow-up	9.66 ± 3.02	9.37 ± 2.70	0.524
	P-value ^a^	0.591	0.052	
Opportunity for use and capacity development	Baseline	6.95 ± 2.42	7.03 ± 2.35	0.841
	Follow-up	7.06 ± 2.42	6.91 ± 2.39	0.692
	P-value ^a^	0.591	0.049	
Total	Baseline	71.22 ± 14.02	71.62 ± 12.61	0.849
	Follow-up	73.27 ± 13.67	70.63 ± 12.73	0.212
	P-value ^a^	0.208	0.054	

Note: a: p-values for comparing scores between the baseline and follow-up (derived from paired t-tests); b: p-values for comparing differences between CG and IG (derived from independent t-test)

*p* < 0.05

*QWL*, quality of life work; *IG*, intervention group; *CG*, control group

The only significant difference between groups was in work and total living space; thus, the mean score of this dimension in CG before and after EP is 8.65 ± 3.04 and 8.05 ± 3.02, respectively, and in IG, the mean score before and after EP is 8.21 ± 2.99 and 11.29 ± 3.93, respectively. Comparing the score of work and total living space in IG before and after EP revealed a statistically significant difference (***p*** = 0.046). In the inter-group comparison, the work and total living space score was not significantly different between the two groups in the stage before EP (*p* = 0.874). Still, this difference became significant three months after EP (***p*** = 0.030). These findings show that improving work and total living space can significantly improve QWL.

## Discussion

The findings of this study revealed that, among the QWL dimensions, work and total living space is one of the educational needs of EMS providers and that by designing EP about QWL based on the educational needs of EMS providers, work and total living space can be improved; however, the total QWL score did not change.

Work and total living space, as one of the primary components of QWL, are critical for both employees and employers. It is difficult to separate family and work life in a competitive environment. Today, most employees express a strong desire to strike a healthy balance between work, family life, and leisure time [32]. This dimension could be equated with work-life balance. Work-life balance refers to the coordination of work and non-work aspects of life and is critical for employees to continue healthy work and for organizations to be sustainable [33]. In most countries, balancing work and personal life is the most important factor for job satisfaction [34]. QWL is a multifaceted entity that is influenced by many aspects of work and personal life. So that, some define the QWL balance between work and family [35].

Unlike in this study, where the EP was designed based on the extracted training needs, Khabbazi Ravandi implemented the EP on QWL for the employees of the Iranian Welfare Organization without conducting a needs analysis, and the results demonstrated that the QWL of the employees of the Welfare Organization can be improved using this EP [18]. Samavatian et al. conducted a study in Ilam, Iran, to investigate the effectiveness of the QWL education program and the enrichment of marital life education on the QWL and job satisfaction of dual-career couples, and the results showed an improvement in QWL and job satisfaction [19].

Non-educational approaches have been used to change QWL in some published studies. In his study, Howe used a team communication program, and the results showed that this type of intervention can improve the dimensions of QWL related to certified nurse aides [36]. Another study looked at the effect of teaching positive thinking through a social media program on nurses’ QWL, and the results showed that the mentioned approach had a positive impact [37]. Huang et al. investigated the effect of Balint group training, which consisted of members of a group presenting a case and having a group discussion about it. They discovered that this approach can improve the QWL of intensive care unit nurses [38].

In general, it should be noted that empowerment and training can help to improve QWL. The findings of Nayak et al. confirmed the same point, demonstrating that empowerment is one of the factors influencing health care employees’ QWL [39].

One of the limitations of this study was providing educational content through the WhatsApp application and uploading the educational content in the form of text and PowerPoint, which may have affected the participants’ learning rate due to the lack of face-to-face education. The second limitation of this study was the participants’ fatigue and inaccuracy in answering the questions. Another was determining educational needs by comparing the mean and median scores, which may have covered only some participants’ needs.

## Conclusion

This study demonstrated that “work and total living space” as one of the QLW dimensions can be improved by designing an EP of QWL based on the educational needs of EMS providers. According to the results of this study, in addition to paying attention to all dimensions of QWL, it is suggested that EMS managers pay more attention to improving EMS providers` work and total living space, including setting the number of shifts and suitable working hours, creating a suitable platform for developing healthy lifestyles, creating opportunities to communicate safely with family while at work and educating employees about healthy lifestyles, and time and stress management.

Future action research studies in EMS should be conducted to study the impact on the quality of working life of EMS providers. It is also proposed to use virtual education supplemented with e-learning techniques for medical professionals in future studies. Although this study was conducted in Iran, it may apply to other countries.

## Data Availability

The data will be made available from the corresponding author for the editor/reviewers upon request.

## Abbreviations

EMS: Emergency medical services
QWL: Quality of work life
EP: Education program
IG: Intervention group
CG: Control group
